# Insufficient NNMT promotes autophagy and disrupts progesterone signaling in endometrial stromal cells in recurrent implantation failure by modulating the H3K9me3-ALDH1A3 pathway

**DOI:** 10.1038/s41420-025-02752-x

**Published:** 2025-10-07

**Authors:** Yifei Song, Shaotong Zhao, Xianping Hou, Jiayuan Chen, Qian Zhang, Shizhen Su, Junhao Yan, Tianxiang Ni

**Affiliations:** 1 State Key Laboratory of Reproductive Medicine and Offspring Health, Center for Reproductive Medicine, Institute of Women, Children and Reproductive Health, Shandong University, Shandong, China; 2 National Research Center for Assisted Reproductive Technology and Reproductive Genetics, Shandong University, Jinan, Shandong China; 3 Key Laboratory of Reproductive Endocrinology (Shandong University), Ministry of Education, Jinan, Shandong China; 4 Shandong Technology Innovation Center for Reproductive Health, Jinan, Shandong China; 5 Shandong Provincial Clinical Research Center for Reproductive Health, Jinan, Shandong China; 6 Shandong Key Laboratory of Reproductive Research and Birth Defect Prevention, Jinan, Shandong China; 7 Research Unit of Gametogenesis and Health of ART-Offspring, Chinese Academy of Medical Sciences (No.2021RU001), Jinan, Shandong China

**Keywords:** Infertility, Autophagy

## Abstract

Defective endometrial receptivity represents an important factor in recurrent implantation failure (RIF), though its precise regulatory mechanisms remain unclear. While nicotinamide N-methyltransferase (NNMT) is abundantly expressed in human endometrial tissues, its role in endometrial receptivity and RIF pathogenesis has not been defined. This study demonstrated that NNMT expression was significantly downregulated in midluteal-phase endometrium from RIF patients relative to fertile controls. Functional analyses in human endometrial stromal cells (ESCs) revealed that NNMT knockdown enhanced autophagy flux and disrupted progesterone signaling. Mechanistically, NNMT deficiency elevated H3K9me3 enrichment at the *Aldh1a3* promoter, suppressing its expression. Notably, knockdown of ALDH1A3 resulted in similar effects with NNMT downregulation, and exogenous rhALDH1A3 reversed the autophagy alterations and rescued progesterone signaling in NNMT-knockdown cells. In vivo, NNMT inhibition in a murine model reduced embryo implantation rates and decreased ALDH1A3 expression. Collectively, these findings indicate that reduced NNMT impairs endometrial receptivity through H3K9me3-mediated ALDH1A3 repression, leading to aberrant autophagy and disrupted progesterone signaling in decidualized ESCs. This study identifies the NNMT-H3K9me3-ALDH1A3 axis as a key epigenetic-metabolic pathway underlying RIF, offering novel diagnostic and therapeutic targets.

## Introduction

Despite the wide application of in vitro fertilisation (IVF) in infertility treatment, approximately 10% of patients fail to achieve clinical pregnancy after multiple high-quality embryo transfers or cumulative transfers of multiple high-quality embryos. This is known as recurrent implantation failure (RIF) [1]. The exact definition of RIF remains controversial, but the most commonly used definition is the one recommended by the European Society of Human Reproduction and Embryology, which defines RIF as ≥3 transfers of high-quality embryos or a cumulative number of ≥10 embryos transplanted without achieving a clinical pregnancy [2].

Endometrial receptivity describes the capacity of the endometrium to accept an embryo. It involves a series of complicated morphological and functional modifications to facilitate blastocyst localisation, adherence, and invasion within a specific window of implantation [3–6]. As a key factor for successful pregnancy, it is affected by abnormal cell biological processes such as differentiation, proliferation, apoptosis, and autophagy [7, 8]. Endometrial receptivity is regulated by ovarian estrogen and progesterone and various transcription factors, cytokines, and signaling pathways [9–11].

Autophagy is a well-preserved cellular degradation process in eukaryotic cells that facilitates the recycling of metabolic materials and the renewal of organelles, ensuring intracellular homeostasis during conditions such as nutrient deficiencies, infections, and hypoxia [12, 13]. In recent years, autophagy has been shown to play an important role in maintaining endometrial homeostasis and normal pregnancy in endometrial tissues [14, 15]. Cyclic alteration of endometrium requires continuous cellular adaptation, which is closely related to autophagy [14]. Abnormal regulation of autophagy occurs in several endometrium-related conditions, such as endometriosis, endometrial hyperplasia, and endometrial cancer, and is positively correlated with their severity [14, 16]. Moreover, a study in 2023 revealed that reduced activation of autophagy in endometrial cells leads to impaired endometrial receptivity and the development of RIF [17]. However, the molecular mechanism by which autophagy regulates endometrial receptivity remains unclear.

The nicotinamide N-methyltransferase gene (NNMT) is localised on human chromosome 11q23.1 and encodes an important methyltransferase [18, 19]. NNMT uses S-adenosyl methionine (SAM) as a methyl donor to catalyse the methylation of nicotinamide and other pyridine derivatives [19]. It is involved in the biotransformation of nicotinamide, drugs, and exogenous compounds and maintains metabolism and energy balance [18, 20]. Recent studies have suggested that abnormal NNMT expression may contribute to the development of cancers, neurological disorders, and obesity by disrupting cellular autophagy [21–23]. Notably, NNMT is highly expressed in human endometrial tissues and has been selected as a biomarker to distinguish mid-secretory endometrium uterine fluid samples of patients with RIF from control samples [24]. However, the regulation of endometrial receptivity by NNMT in RIF and the associated mechanism have not been discovered.

In this study, we found reduced NNMT gene expression levels in the midluteal-phase endometrium in patients with RIF compared to control patients. Using an immortalized human endometrial stromal cell line (THESCs) and primary human endometrial stromal cells (ESCs), NNMT knockdown was shown to interfere with progesterone signaling and promote autophagy by suppressing ALDH1A3, which impaired endometrial receptivity. Exogenous supplementation of recombinant human ALDH1A3 protein partially rescued the cellular phenotype. Mechanistic studies revealed that reduced NNMT levels increased the levels of H3K9me3, an inhibitory histone modification, and led to greater enrichment of H3K9me3 in the ALDH1A3 promoter region. In vivo, selective inhibition of NNMT inhibited embryo implantation and ALDH1A3 expression of implantation sites. Collectively, our findings demonstrated a key contribution of insufficient NNMT to the development of RIF through the modulation of the H3K9me3-ALDH1A3 axis, providing a useful complement to the regulatory mechanism of endometrial receptivity and new insights into the prevention and treatment of RIF.

## Results

### NNMT expression levels were low in the midluteal-phase endometrium of patients with RIF but increased with decidualization

**Fig. 1:**
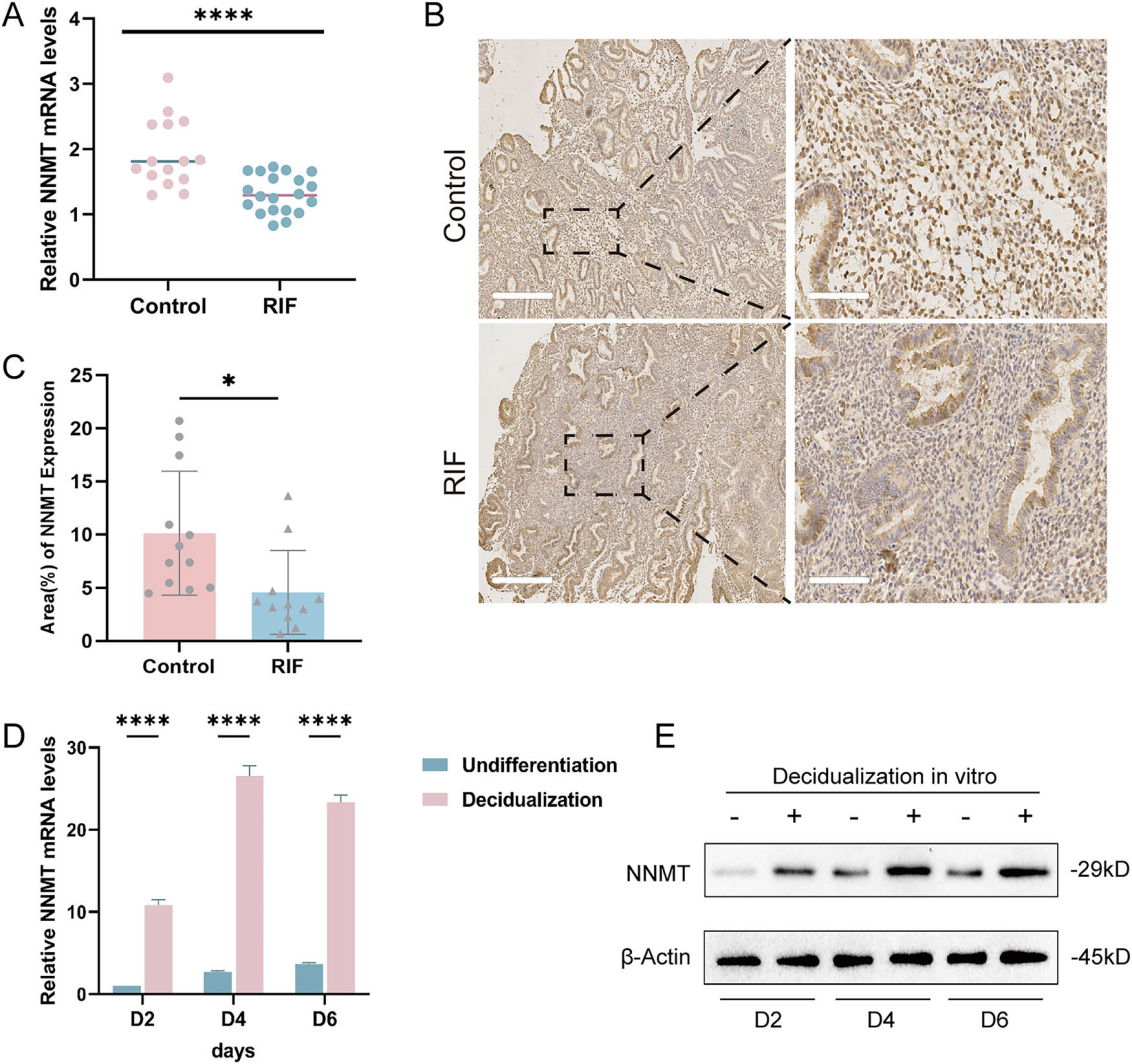
NNMT expression levels were low in the midluteal-phase endometrium of patients with RIF but increased with decidualization. **A** qPCR analysis for NNMT mRNA levels in midluteal-phase endometrial tissues of Control and RIF patients; **B**, **C** Immunohistochemistry analysis for NNMT expression in midluteal-phase endometrial tissues of Control and RIF patients (scales for 400 μm and 80 μm, respectively); **D** qPCR analysis for NNMT mRNA levels of THESCs with or without induced decidualization; **E** Western blot analysis for NNMT protein levels of THESCs with or without induced decidualization; Data are presented as the mean ± SD, *: *p* < 0.05, ****: *p* < 0.0001.

**Fig. 2:**
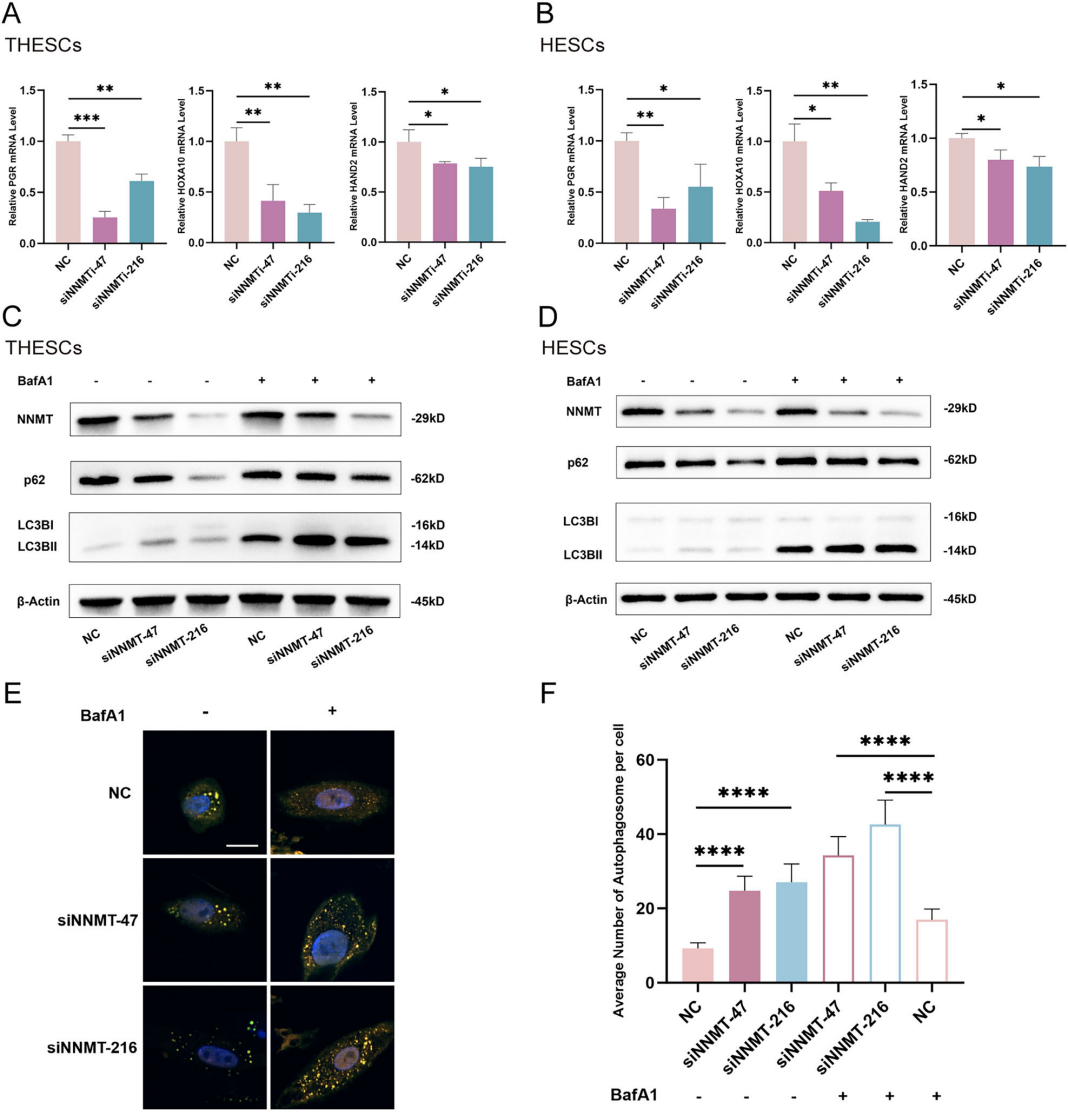
NNMT knockdown promoted autophagy and impaired progesterone signaling during decidual process. **A**, **B** qPCR analysis for *PGR*, *HOXA10,* and *HAND2* mRNA levels of THESCs and HESCs in NC and knockdown groups after induced decidualization for 4 days; **C**, **D** Western blot analysis for p62 and LC3B II protein levels of THESCs and HESCs in NC and knockdown groups after induced decidualization; **E**, **F** mCherry-eGFP-LC3 fluorescence autophagy experiment for observing autophagosomes of THESCs in NC and knockdown groups after induced decidualization (scale for 15 μm); Data are presented as the mean ± SD, *: *p* < 0.05, **: *p* < 0.01, ***: *p* < 0.001, ****: *p* < 0.0001.

Initially, we used qPCR to verify the expression of NNMT in midluteal-phase endometrial samples obtained from 15 control patients and 20 patients with RIF. The results showed that NNMT levels were significantly lower in patients with RIF than in fertile women (Fig. 1A). Subsequently, we validated this result by showing NNMT protein level differences using immunohistochemical staining of endometrial tissues from 12 control patients and 11 patients with RIF (Fig. 1B, C). To investigate the expression of NNMT during the decidualization process in ESCs, we extracted mRNA and protein from decidualized and untreated THESCs at D2, D4, and D6. Immunoblotting and qPCR analyses showed that NNMT levels increased following decidualization, and the difference at D4 was most evident (Fig. 2D, E). Therefore, decidualized THESCs at D4 were used for subsequent experiments. These results suggested that NNMT expression was upregulated in decidualized THESCs and downregulated in midluteal-phase endometrial tissues of patients with RIF, which implied that NNMT might play a crucial role in the pathogenesis of RIF.

### NNMT knockdown promoted autophagy and impaired progesterone signaling during decidual process

To investigate the role of NNMT in human ESCs, we designed two siRNAs (siNNMT-47 and siNNMT-216). We first verified the knockdown efficiency in THESCs after transfection. The results demonstrated a significant reduction of NNMT RNA and protein levels (Fig. S1A–D).

To determine the effects of NNMT knockdown on the functions of ESCs, we measured the mRNA levels of *PRL* and *IGFBP1*, which are decidualization markers, and *PGR*, *HOXA10*, and *HAND2*, which are related to the progesterone signaling pathway at D4 of decidualization induction in THESCs and HESCs. The results showed that *PGR, HOXA10*, and *HAND2* levels significantly decreased in the knockdown group compared with the negative control group, while *PRL* and *IGFBP1* levels were similar between groups, suggesting that NNMT knockdown impaired progesterone signaling in ESCs but did not affect cAMP-mediated decidualization pathways (Figs. 2A, B and S1E, F).

Furthermore, it has been reported that the absence of the key autophagy protein FIP200 mediates the decidualization failure of stromal cells via aberrant progesterone signaling. Progesterone can also activate autophagy flux via the MAPK/ERK pathway. Therefore, we subsequently detected the alteration of autophagic flux in stromal cells following NNMT knockdown. We detected the autophagy markers LC3B and P62 using immunoblotting in THESCs and HESCs after NNMT knockdown and decidualization induction for 4 days, combined with treatment with or without the autophagy inhibitor bafilomycin A (BafA1). The results showed that the expression level of P62 was reduced, while the expression level of LC3B II increased in both THESCs and HESCs after NNMT knockdown regardless of BafA1 treatment (Fig. 2C, D). mCherry-eGFP-LC3 adenovirus infection and confocal microscopy were used to observe autophagosomes in THESCs. Consistent with the immunoblotting results, more LC3 puncta appeared in NNMT-knockdown cells than negative control cells (Fig. 2E, F). These results suggested that reduced NNMT levels may lead to enhanced autophagy in decidualized stromal cells.

### NNMT knockdown suppressed the transcription of ALDH1A3 by increasing H3K9me3 levels

**Fig. 3:**
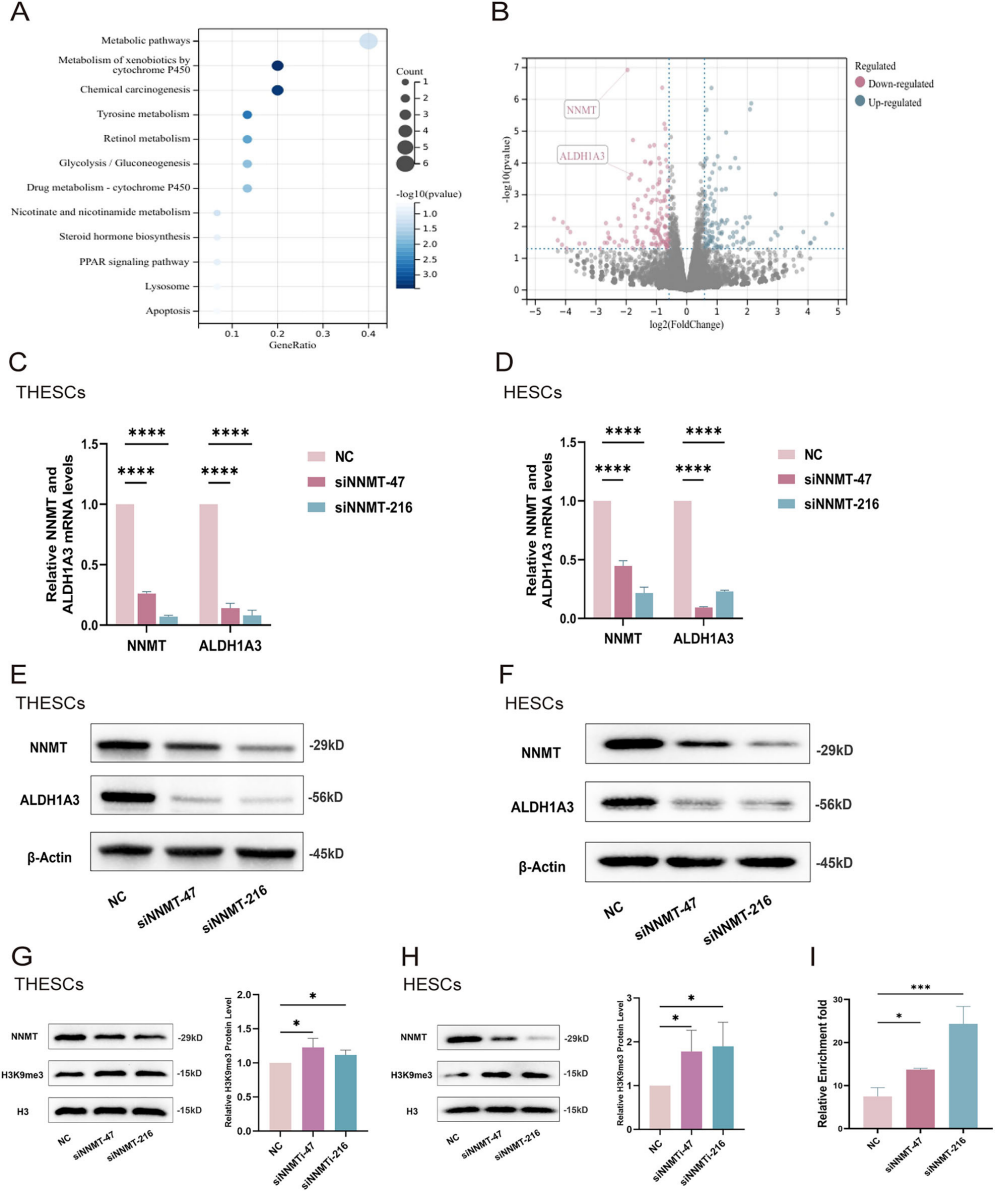
NNMT knockdown suppressed the transcription of ALDH1A3 by increasing H3K9me3 levels. **A** KEGG (Kyoto Encyclopedia of Genes and Genomes) analysis for differentially expressed genes of THESCs between the NC and knockdown groups after induced decidualization for 4 days as determined by RNA-seq analysis; **B** Volcano plot for differentially expressed genes of THESCs between the NC and knockdown groups after induced decidualization as determined by RNA-seq analysis; **C**, **D** qPCR analysis for NNMT and *ALDH1A3* mRNA levels of THESCs and HESCs in the NC and knockdown groups after induced decidualization; **E**, **F** Western blot analysis for NNMT and ALDH1A3 protein levels of THESCs and HESCs in NC and knockdown groups after induced decidualization; **G**, **H** Western blot analysis for H3K9me3 protein levels of THESCs and HESCs in NC and knockdown groups after induced decidualization; **I** CUT&RUN analysis for binding degree of H3K9me3 to the promoter region of *ALDH1A3* gene of THESCs in NC and knockdown groups after induced decidualization; Data are presented as the mean ± SD, *: *p* < 0.05, ***: *p* < 0.001, ****: *p* < 0.0001.

To examine potential molecular targets of NNMT in human stromal cells, we used RNA sequencing to profile the transcriptomes of NNMT-knockdown THESCs in the decidualization state. Based on an analysis of the sequencing data, *ALDH1A3*, which encodes a retinaldehyde-metabolising enzyme that is critical for retinoic acid biosynthesis, implicated in stem cell differentiation and the oxidative stress response, and thus associated with cancers and metabolic disorders, was significantly downregulated after NNMT knockdown (Fig. 3A, B). The expression of ALDH1A3 was verified in THESCs and HESCs after NNMT knockdown through qPCR and immunoblotting (Fig. 3C–F).

NNMT catalyses the irreversible transfer of the methyl group from SAM to nicotinamide, thus altering a wide variety of metabolic processes such as methionine cycles, chromatin remodelling, and histone (including H3K9) methylation [18]. Therefore, we further investigated the impact of NNMT knockdown on H3K9me3 levels. The immunoblotting results showed that the H3K9me3 levels in NNMT-knockdown groups increased on D4 of decidualization compared to negative control group (Fig. 3G, H).

Considering that H3K9me3 modification in promoter regions generally suppresses the transcription of target genes, we hypothesised that NNMT knockdown might inhibit *ALDH1A3* transcription by increasing H3K9me3 levels. Therefore, we searched the ENCODE database (www.encodeproject.org) and found that the *ALDH1A3* promoter region harboured an H3K9me3 modification site, which supported the above-mentioned hypothesis. Further, we performed CUT&RUN assays to monitor the interaction between H3K9me3 and the promoter region of the *ALDH1A3* gene after NNMT knockdown. Knockdown of NNMT resulted in increased H3K9me3 enrichment at the *ALDH1A3* promoter region (Fig. 3I). In summary, these findings suggest that aberrant expression of the *NNMT* gene may suppress the expression of *ALDH1A3* by elevating H3K9me3 levels.

### ALDH1A3 mediated NNMT-induced autophagy and decidualization

To further investigate the role of ALDH1A3 in human ESCs, we designed an siRNA (siALDH1A3) and verified the efficacy of knockdown of its target at the RNA and protein level (Fig. S1G–J).

**Fig. 4:**
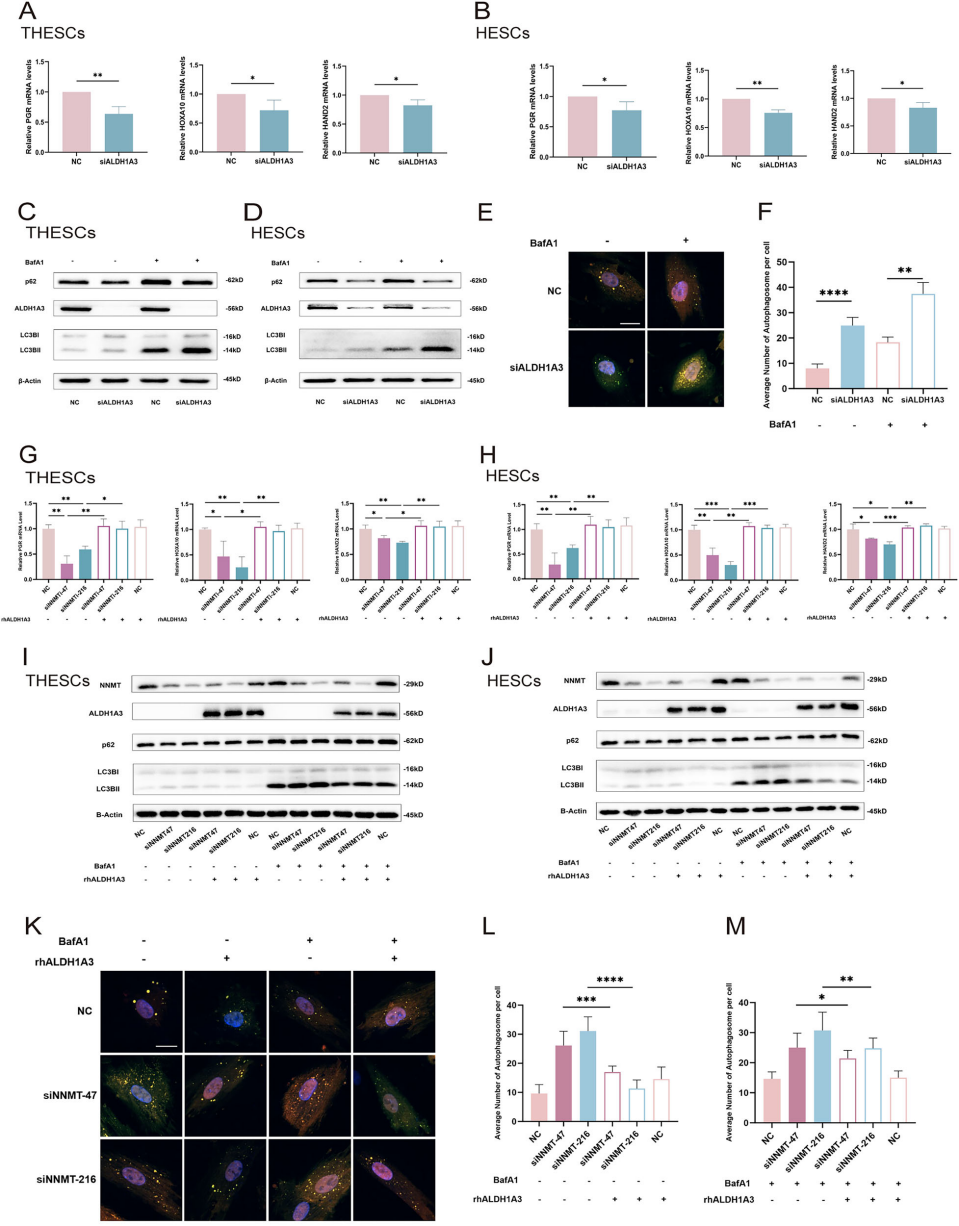
ALDH1A3 mediated NNMT-induced autophagy and decidualization. **A**, **B** Western blot analysis for p62 and LC3B II protein levels of THESCs and HESCs in NC and siALDH1A3 groups after induced decidualization for 4 days; **C**, **D** mCherry-eGFP-LC3 fluorescence autophagy experiment for observing autophagosomes of THESCs in NC and siALDH1A3 groups after induced decidualization (scale for 15 μm); **E**, **F** qPCR analysis for *PGR*, *HOXA10,* and *HAND2* mRNA levels of THESCs and HESCs in NC and siALDH1A3 groups after induced decidualization; **G**, **H** Western blot analysis for p62 and LC3B II protein levels of THESCs and HESCs in NC and knockdown groups after induced decidualization with or without rhALDH1A3; **I**–**K** mCherry-eGFP-LC3 fluorescence autophagy experiment for observing autophagosomes of THESCs in NC and knockdown groups after induced decidualization with or without rhALDH1A3 (scale for 15 μm); **L**, **M** qPCR analysis for *PGR*, *HOXA10,* and *HAND2* mRNA levels of THESCs and HESCs in NC and siNNMT groups with or without rhALDH1A3 after induced decidualization; Data are presented as the mean ± SD, *: *p* < 0.05, **: *p* < 0.01, ***: *p* < 0.001, ****: *p* < 0.0001.

Four days after transfection and induction of decidualization in vitro, ALDH1A3 knockdown decreased the expression levels of *PGR*, *HOXA10*, and *HAND2* in THESCs and HESCs (Fig. 4A, B), without altering the expression levels of *PRL* or *IGFBP1* (Fig. S1K, L), which is consistent with the effect of NNMT reduction. Similarly, ALDH1A3 knockdown suppressed the expression of p62 and promoted the expression of LC3BII in THESCs and HESCs (Fig. 4C, D), and the mCherry-eGFP-LC3 fluorescence assay showed an increase in the number of autophagosomes in THESCs after ALDH1A3 knockdown, with autophagic flow inhibited after 4 h of BafA1 treatment (Fig. 4E, F). In summary, the reduced expression of *ALDH1A3*, a target gene of NNMT, also inhibited progesterone signaling and promoted autophagy in ESCs.

We further investigated whether supplementation with exogenous human recombinant ALDH1A3 (rhALDH1A3) could rescue autophagy and progesterone signaling in THESCs and HESCs following the downregulation of NNMT. Exogenous rhALDH1A3 was shown to improve progesterone signaling in THESCs and HESCs decreased by siNNMT (Fig. 4G, H). Moreover, the western blotting and mCherry-eGFP-LC3 fluorescence experiments showed that in the cells with NNMT knockdown and induced decidualization, the addition of rhALDH1A3 partially rescued autophagy (Fig. 4I–M).

### NNMT inhibition impaired implantation in mice

**Fig. 5:**
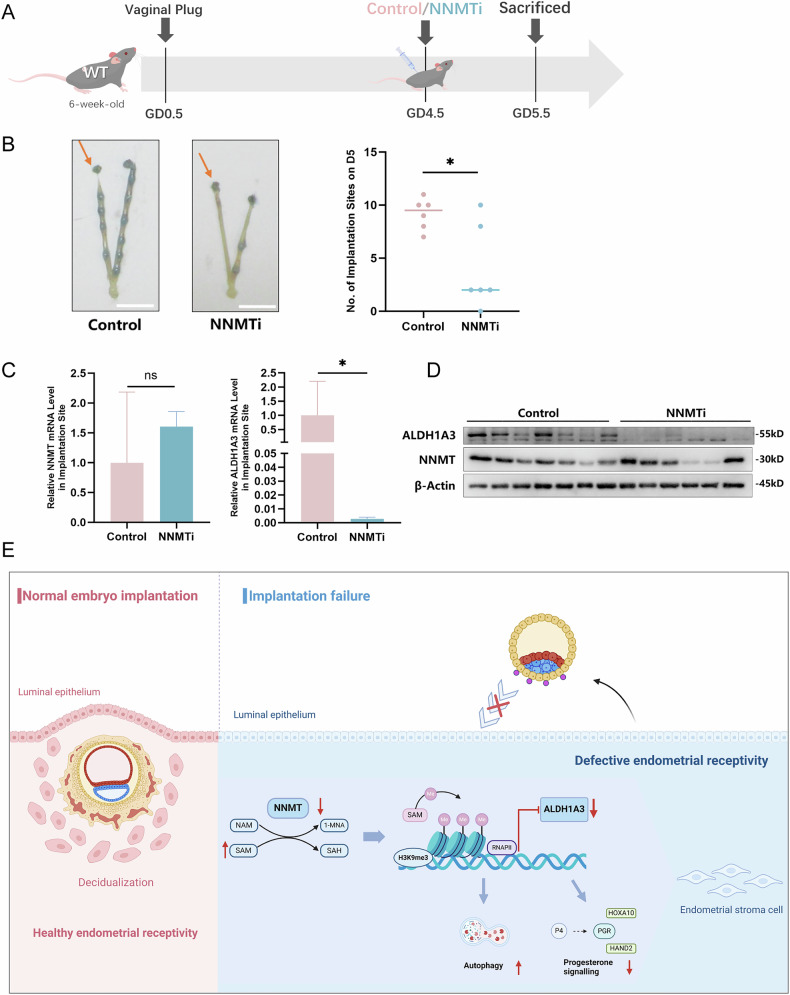
NNMT inhibition impaired implantation in mice. **A** Step chart about animal experiments; **B** On GD5.5 the uterine implantation sites were counted and collected for qPCR and Western blot (scale for 1 cm); **C**, **D** qPCR and Western blot for NNMT and ALDH1A3 mRNA and protein levels of implantation sites in NNMTi group and Control group; **E** Mechanistic modeling of nicotinamide N-methyltransferase in recurrent implantation failure; Data are presented as the mean ± SD, *n* = 6 mice/group, *: *p* < 0.05.

To explore the effects of NNMT knockdown on embryo implantation in vivo, we used wild-type C57BL/6 mice to create a uterine NNMT inactivation group (NNMTi group) and a control group by unilateral horn injection of a selective NNMT inhibitor or a blank solvent, respectively, at GD4.5. At GD5.5, pregnant mice were sacrificed, and the implantation sites were visualised and recorded using a Chicago blue dye solution (Fig. 5A). The results indicated that there were significantly fewer implantation sites in the NNMTi group than the control group (Fig. 5B). Additionally, decreased expression levels of ALDH1A3 in the NNMTi group compared with the control group were observed using qPCR and immunoblotting assays (Fig. 5C, D). These in vivo findings verified that NNMT inhibition led to implantation failure by modulating ALDH1A3.

## Discussion

The establishment of endometrial receptivity plays an essential role in the pathogenesis of RIF, but the molecular mechanism remains largely unknown. Our results demonstrated that reduced NNMT expression levels in the midluteal-phase endometrium promoted autophagy and interfered with progesterone signaling in ESCs during decidualization through the H3K9me3-ALDH1A3 pathway, which ultimately impaired endometrial receptivity and contributed to RIF (Fig. 5E).

NNMT is a methyltransferase that catalyses the methylation of nicotinamide (NAM) by utilising SAM as the methyl donor, linking single-carbon metabolism directly to cellular methylation homeostasis and nicotinamide adenine dinucleotide (NAD+) [25]. Previous studies have reported that the expression of NNMT was upregulated in the mid-secretory endometrium compared with the proliferative endometrium [24]. Meanwhile, it was discovered that NNMT is regulated by progesterone and its expression is significantly downregulated in the endometrium of patients with RIF [26]. The reduction of NNMT could distinguish between the mid-secretory phase of the endometrium in fertile women and women with RIF [24]. In addition, significant differences were found in the expression levels of NNMT in the receptive endometrium between patients who had successful pregnancies after ICSI cycles and those who were still not pregnant after at least two ICSI cycles [27]. Our findings was consistent with previous findings, confirming that the expression of NNMT is reduced in endometrial tissues during the window of implantation among patients with RIF, suggesting its potential role in the pathogenesis of RIF.

Autophagy is a catabolic process prevalent in eukaryotic cells, and it plays an important role in the homeostasis of cells, tissues, and organisms [28–30]. Many studies have substantiated the role of autophagy in tumor formation, and the process of embryonic invasion into the uterus has some similarities with tumorigenesis [31, 32]. Previous studies have found that autophagy is involved in the maintenance of normal endometrial function during the peri-implantation period and plays an important role in endometrial receptivity, stromal cell decidualization, and embryo implantation [33, 34]. Young Choi’s group discovered that the mouse uterus shows a heightened autophagic response on days 1 and 2 when it experiences an inflammatory reaction after mating. However, this response diminishes when implantation occurs [35]. A study published in 2023 found that activation of NOTCH1-pathway-mediated autophagy in endometrial cells can impair endometrial receptivity, leading to RIF [17]. However, it was also found that reduced or absent autophagy can affect endometrial growth and receptivity through mTOR-related or non-mTOR-related pathways, which may contribute to infertility and RIF [7, 36, 37]. It is still controversial whether increased or decreased autophagy levels contribute to RIFs. Deficient autophagy disrupts cellular metabolic homeostasis, while excessive autophagy results in uncontrolled self-digestion of essential cellular components, ultimately triggering autophagic cell death and tissue damage [38, 39]. Meanwhile, the effect of NNMT on autophagy has also been probed in previous studies. Recent studies have reported that the depletion of NNMT promotes hepatocellular carcinoma cell survival by enhancing autophagy under conditions of starvation [21]. The authors of a study conducted in 2020 discovered that NNMT hinders the process of oxidative stress-induced autophagy in breast cancer cells by inhibiting the AMPK pathway [23]. Some studies have also found a close relationship between NNMT and autophagy in neural and adipose tissues [22, 40]. From the perspective of the modulatory effects of autophagy on endometrial receptivity, our findings revealed that insufficient NNMT was closely associated with a high level of autophagy in ESCs, which might aggravate cell damage and consequently impair cell decidualization and endometrial receptivity. However, the precise molecular mechanisms by which NNMT and ALDH1A3 modulate autophagy-related proteins remain poorly understood and warrant further investigation.

We also found that reduced NNMT levels affected progesterone signaling in ESCs and impaired endometrial receptivity. Endometrial receptivity occurs for 6–10 days following ovulation and is modulated by estrogen and progesterone. The role of progesterone signaling in normal pregnancy establishment has been elaborately studied [41]. Molecules such as PGR, along with its downstream targets HOXA10 and HAND2, are crucial for the decidualization of ESCs and the formation of a receptive endometrium [42]. Previous studies by Lydon et al. using the *Pgr*-knockout mouse model found that nuclear receptor-mediated progesterone signaling is essential for the establishment of endometrial decidualization and receptivity [43]. HOXA10 has important functions in regulating endometrial development during the menstrual cycle and embryo implantation [44]. *Hoxa10* mutations have been reported to cause specific stromal cell defects, resulting in defective endometrial decidualization and abnormal embryo implantation [45]. In addition, HAND2 is a progesterone-induced transcription factor in the endometrial stroma and is associated with endothelial receptivity and decidualization [46]. Moreover, mice with uterine deletion of Hand2 exhibit impaired embryo implantation [47]. Our findings indicated that decreased NNMT levels led to a substantial decrease in the mRNA levels of *PGR*, *HOXA10*, and *HAND2*, therefore affecting progesterone signaling.

In the preliminary stage of this study, we also investigated the expression levels of *PRL* and *IGFBP1*, which are the most commonly used metamorphosis markers for decidualization, and found no significant differences, indicating that NNMT regulated the decidualization of ESCs not through the cAMP signaling pathway, but rather via a PGR-mediated progesterone signaling cascade.

*ALDH1A3* encodes an aldehyde dehydrogenase enzyme that is critical for retinoic acid biosynthesis and has been implicated in stem cell differentiation and the oxidative stress response and associated with cancers and metabolic disorders. *ALDH1A3* has been reported to be aberrantly expressed in a variety of malignant tumors and to be closely associated with metabolism in previous studies [48–51]. Moreover, a significant difference in the expression level of ALDH1A3 in the endometrial receptive stage has been reported between patients with successful pregnancies after ICSI cycles and patients who do not achieve a pregnancy after at least two ICSI cycles [27]. However, its role in the uterus has not been fully investigated. Our findings revealed that ALDH1A3 contributes to the regulation of endometrial receptivity through its modulatory effects on autophagy and decidualization processes, thereby shedding new light on its regulatory role during the window of implantation.

There is no direct evidence of an interaction between NNMT and ALDH1A3. However, potential cross-regulation at the pathway level cannot be ruled out. Specifically, it has been reported that NNMT activates the PI3K/AKT signaling pathway via TTPAL, by which the expression of ALDH1A3 is modified [52, 53].

Given that NNMT is an important methyltransferase, changes in its expression level can cause more or fewer methyl groups of SAM to be used for the methylation of target molecules, such as histones or DNA [54]. Previous research has indicated that high expression levels of NNMT deplete excess reactive methyl groups, leading to a decrease in the H3K4me3 modification of the E-cadherin promoter and suppression of the m6A modification of E-cadherin mRNA. As a result, the expression of E-cadherin is inhibited at both the transcriptional and post-transcriptional levels [55]. In addition, Couto et al. discovered that reduced NNMT levels trigger an excess of methyl groups, resulting in an increase in histone H3K9 trimethylation and DNA methylation at the promoters of genes related to the extracellular matrix [56]. Meanwhile, several scholars have demonstrated that the transcription of *ALDH1A3* can be activated by H3K27ac and repressed by H3K9me3 in its promoter region, which is consistent with our findings [57, 58]. Our results demonstrated that downregulated NNMT levels increased the levels of H3K9me3, an inhibitory histone modification, and led to the enrichment of H3K9me3 in the *ALDH1A3* promoter region, resulting in its reduced transcription level.

## Conclusions

In summary, our findings from in vitro and in vivo studies revealed that decreased NNMT levels in the midluteal-phase endometrium of patients with RIF interfered with progesterone signaling and promoted the autophagy of ESCs through the H3K9me3-ALDH1A3 pathway, which ultimately impaired endometrial receptivity and contributed to RIF. Our findings enrich our knowledge of the etiological mechanism of RIF from autophagic and epigenetic perspectives and provide new targets for predicting endometrial receptivity. This is important for the precise prevention and treatment of RIF and for improving the success rate of IVF.

## Materials and methods

### Sample collection from RIF patients and controls

Patients were assigned to the RIF group if they met the following criteria: (1) they did not achieve a successful clinical pregnancy after ≥ 3 fresh or frozen embryo transfers, or a total of ≥ 4 high-quality embryos; (2) they and their partner had no chromosomal abnormalities; (3) they had no uterine structural abnormalities, space-occupying lesions, or endometritis; (4) they had no endocrine or autoimmune disorders; and (5) they had not undergone an intrauterine operation or received hormonal drugs in the past 7 days.

Patients were assigned to the control group if they met the following criteria: (1) they had previously given birth to a healthy baby; (2) they underwent IVF/intracytoplasmic sperm injection (ICSI) because of a male factor or female fallopian tube factor; (3) they and their partner had no chromosomal abnormalities; (4) they had no uterine structural abnormalities, space-occupying lesions, or endometritis; (5) they had no endocrine or autoimmune disorders; (6) they had no history of complications during pregnancy or delivery, such as embryo implantation failure or spontaneous miscarriage; and (7) they did not undergo an intrauterine operation or receive hormonal drugs in the past 7 days. The patients enrolled in the study were monitored for ovulation using ultrasonography, and an endometrial biopsy was performed on days 7–9 after ovulation.

This study was approved by the Ethics Committee of the Reproductive Medicine Centre of Shandong University, and informed consent was obtained from all patients.

### Animal experiments

All mice involved in experiments were wild-type C57BL/6 mice purchased from Gempharmatech (Jiangsu, China) and bred in a temperature-controlled chamber under a constant 12 h/12 h light/dark cycle in the Model Animal Research Centre of Shandong University (Jinan, China) according to the National Institutes of Health and institutional guidelines for the use and care of laboratory animals. The day when a vaginal plug was observed after 6-week-old female mice were mated overnight was defined as gestation day 0.5 (GD0.5).

NNMTi (HY-131042; MedChemExpress, Monmouth Junction, NJ, USA) functions through its quinolinium scaffold to selectively bind to the NNMT substrate-binding site through a combination of hydrophobic, hydrogen-bonding, and electrostatic interactions [59]. On GD4.5, pregnant mice were anaesthetised and then randomly injected with NNMTi (1 mg/mouse) or blank solvent into the right uterine horn through a dorsal incision. In this way, we built a mouse model with localised NNMT inactivation in the uterus before embryo implantation (NNMTi group) with no blinding. The sample size for the NNMTi group and the control group was both six. On GD5.5, we anesthetised the mice with isoflurane and visualised the implantation sites using a Chicago Blue dye solution (200 μL/mouse) injected intravenously. The mice were then sacrificed by euthanasia with carbon dioxide. The number and morphology of implantation sites were recorded, and uterine tissues were collected for further quantitative experiments.

### Cell culture and treatment

The THESCs (ATCC CRL-4003) were generously supplied by Professor H. B. Wang from Xiamen University, China. The HESCs were obtained from proliferative-phase endometrial tissues of fertile patients with no disease or hormonal medication. The cells remained undifferentiated when the ESCs were cultured in phenol-red-free DMEM/F12 medium (M&C Gene Technology, Beijing, China) containing 10% charcoal-stripped fetal bovine serum (CS-FBS), 1% NaHCO3, and 1% penicillin/streptomycin (full medium). To induce in vitro decidualization, 0.5 mM db-cAMP (cAMP; Sigma, St. Louis, MO, USA) and 1 mM medroxyprogesterone acetate (Sigma) were added to phenol-red-free DMEM/F12 medium containing 2% CS-FBS, 1% NaHCO3, and 1% penicillin/streptomycin (differentiation medium) for 2–4 days. The medium was changed every 48 h. The ESCs were treated with 50 nM Baf-A1 (Selleck, Houston, TX, USA) for 4 h to inhibit autophagy.

### Transfection

Transfection was performed on the second day after seeding the cells onto plates. The small interfering RNA (siRNA; Boshang, Shandong, China) was combined with Lipofectamine® RNAiMAX (13778150; Invitrogen, Waltham, MA, USA) and Opti-MEM® serum-reduced medium (31985070; Gibco, Waltham, MA, USA) according to the manufacturer’s instructions, and the mixture was incubated at 10 °C–30 °C for 25 min. Then the transfection complex was carefully added to the wells that contained culture medium without antibiotic and mixed gently. After 6 h of transfection, the medium was substituted with either full or differentiation medium.

### RNA extraction and real-time quantitive PCR

Following the manufacturer’s directions, total RNA was isolated from cells or tissues using TRIzol (Invitrogen). The purity and concentration of the extracted RNA samples were measured using a Nanodrop 2000 instrument (Thermo Fisher Scientific, Waltham, MA, USA). A PrimeScript RT reagent kit with gDNA Eraser (TaKaRa, Kusatsu, Japan) was used for reverse transcription of the RNA to cDNA. Green™ Premix Ex Taq™ (TaKaRa) was used for quantitative PCR (qPCR) on a LightCycler 480 II instrument (Roche, Basel, Switzerland). The primers were synthesised by Boshang Biotech (Shandong, China). All expression levels were normalised to β-actin levels. The fold change between different groups was calculated using the 2 − ΔΔCT method.

### Western blot (WB)

Cells were lysed with 1× sodium dodecyl sulphate (SDS) (Beyotime, Shanghai, China) containing 1% protease and phosphatase inhibitor cocktail (Cell Signaling Technology, Danvers, MA, USA). Protein samples were mixed with 5× loading buffer (Beyotime), boiled for 10 min at 100 °C, and then stored at −80 °C. Proteins were separated using 12% SDS-polyacrylamide gel electrophoresis and then transferred to 0.22-μm polyvinylidene fluoride (PVDF) membranes (Millipore, Burlington, MA, USA). Following blocking with 5% non-fat milk for 1 h and subsequent washing with Tris-buffered saline containing Tween 20, the PVDF membranes were incubated with primary antibodies, including anti-NNMT (ab119758; Abcam, Cambridge, UK), anti-PCNA (sc-53407, Santa Cruz, Dallas, TX, USA), anti-LC3B (L7543, Sigma), anti-p62 (ab109012, Abcam), anti-ALDH1A3 (25167-1-AP; Proteintech, Hubei, China), anti-H3K9me3 (07-442, Sigma), anti-H3 (ab1791, Abcam), and anti-β-actin (4970S, Cell Signaling Technology) antibodies, at 4 °C overnight. The next day, the membranes were washed and incubated with secondary antibodies conjugated to horseradish peroxidase (Proteintech) at 10 °C–30 °C for 1 h. Protein bands on the immunoblots were detected using an enhanced chemiluminescence kit (Millipore). The protein bands were analysed using Image Lab software (Bio-Rad, Hercules, CA, USA).

### Immunohistochemistry

Endometrial tissues collected during the midluteal-phase were fixed with 4% paraformaldehyde for 12 h at 10 °C–30 °C and then embedded in paraffin. Following sectioning of the tissues into 4-μm slices, xylene and a graded ethanol series were employed to deparaffinise and rehydrate the samples. The tissue sections were then blocked with 10% bovine serum albumin and 0.3% Triton ×-100. Subsequently, they were incubated with an anti-NNMT antibody (ab119758, Abcam) overnight, and then incubated with biotinylated goat anti-rabbit IgG (Proteintech). After washing with phosphate-buffered saline, the chromogenic reagent 3,3′-diaminobenzidine (ZSGB-BIO, Beijing, China) was applied for staining. Finally, the sections were observed and photographed using an inverted microscope (Leica, Wetzlar, Germany) and quantified using Image J software (National Institutes of Health, Bethesda, MD, USA).

### mCherry-eGFP-LC3 fluorescence assay

siRNA transfection was performed on the day after spreading the 24-well crawler seed plates (801010; NEST, Jiangsu, China). Six hours later, the cells were infected with an mCherry-eGFP-LC3 adenovirus (HanBio Biotechnology, Shanghai, China) diluted with differentiation medium, and another 6 h later, the medium was changed to differentiation medium. On day 4 after transfection, the cells were incubated with BafA1 for 4 h before the coverslips were dipped in 4% paraformaldehyde for 10 min and sealed with DAPI-containing mounting medium. Images were captured using a confocal microscope (Leica) and quantified using Image J software.

### CUT&RUN

On day 4 after siRNA transfection, the cells were collected, separated by centrifugation to extract the sediment, and then reconstituted by adding wash buffer (HD101-01; Vazyme Biotech, Jiangsu, China). The cells were then incubated with ConA Beads Pro at 10 °C–30 °C for 10 min. Eppendorf tubes containing the cells were positioned on a magnetic frame and the supernatant was discarded. The pre-cooled antibody buffer, anti-H3K9me3 (Abcam), and IgG (Millipore) in each sample was then allowed to stand overnight at 4 °C. On the next day, the Dig-wash Buffer was added to the Eppendorf tubes. The tubes were then positioned on a magnetic frame and the supernatant was discarded. Subsequently, the pG-MNase enzyme premix was added to the samples and the samples were rotated at 4 °C for 1 h. They were then incubated with CaCl_2_ premix on ice for 1 h to fragment the DNA. Each sample was then treated with Stop Buffer to release and terminate the DNA fragmentation reaction. Buffer GDP and Buffer GW were added to the samples to extract the DNA, and the extracted products were stored at −20 °C for subsequent qPCR analysis (within 1 week).

### Statistical analysis

Graphs were created using GraphPad Prism 9 (GraphPad Software, San Diego, CA, USA) and Adobe Illustrator 23 (Adobe, San Jose, CA, USA). Statistical analyses were conducted using SPSS version 20.0 (IBM, Armonk, NY, USA). Data that followed a normal distribution are shown as the mean ± standard deviation. Statistical differences were assessed using the two-tailed Student’s *t*-test. Conversely, data that did not follow a normal distribution are represented as medians and quartiles, and statistical differences were examined using the Mann–Whitney *U* test. The difference was considered to be statistically significant when *p* < 0.05. Moreover, all experimental groups in this study included at least three independent biological replicates per assay.

## Supplementary information

Supplementary legends

Original images of western blot

Figure S1

Original datas

## Data Availability

Data that support the findings of this study are available from the corresponding author upon reasonable request.
