# Supplementary material for: Insufficient NNMT promotes autophagy and disrupts progesterone signaling in endometrial stromal cells in recurrent implantation failure by modulating the H3K9me3-ALDH1A3 pathway
Source: Cell Death Discov. 2025 Oct 7;11:450. doi: 10.1038/s41420-025-02752-x (PMC12504709; doi:10.1038/s41420-025-02752-x)
Supplement: Supplementary file 1 — Supplementary legends [file 41420_2025_2752_MOESM1_ESM.docx]

**Supplementary legends**

**Figure S1.** (A, B) qPCR analysis for NNMT mRNA levels of THESCs and HESCs; (C, D) Western blot analysis for NNMT protein levels of THESCs and HESCs; (E, F) qPCR analysis for PRL and IGFBP1 mRNA levels of THESCs and HESCs after induced decidualization for 4 days; (G, H) qPCR analysis for ALDH1A3 mRNA levels of THESCs and HESCs; (I, J) Western blot analysis for NNMT protein levels of THESCs and HESCs; (K, L) qPCR analysis for PRL and IGFBP1 mRNA levels of THESCs and HESCs after induced decidualization for 4 days.
