# Supplementary material for: Insufficient NNMT promotes autophagy and disrupts progesterone signaling in endometrial stromal cells in recurrent implantation failure by modulating the H3K9me3-ALDH1A3 pathway
Source: Cell Death Discov. 2025 Oct 7;11:450. doi: 10.1038/s41420-025-02752-x (PMC12504709; doi:10.1038/s41420-025-02752-x)

**
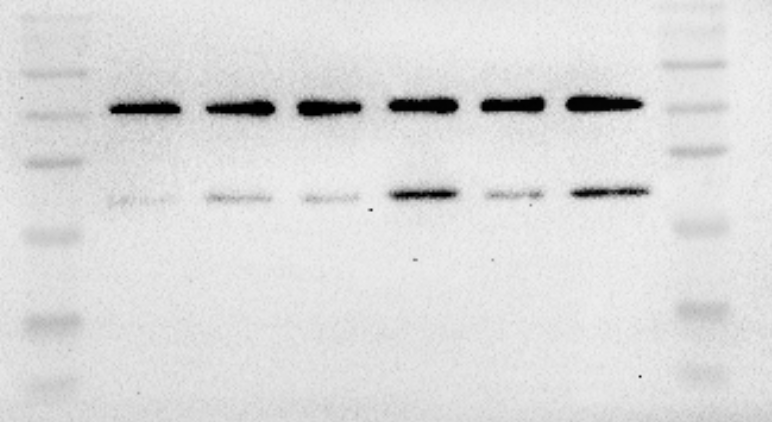
（1）Figure 1E**

-29kD

-45kD

β-Actin

NNMT

**（2）Figure 2C**


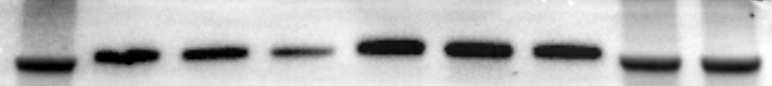

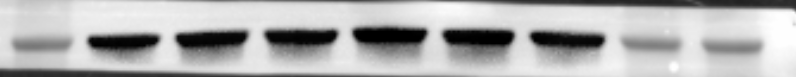

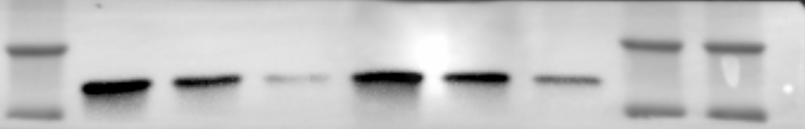

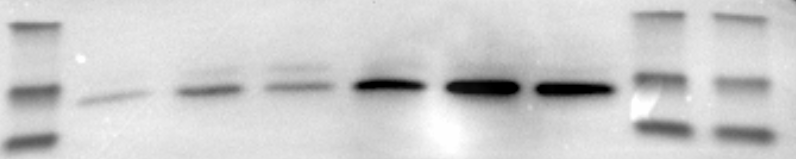


β-Actin

NNMT

-45kD

-29kD

P62

-62kD

LC3B I

LC3B II

-16kD

-14kD

**（3）Figure 2D**


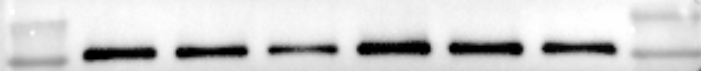

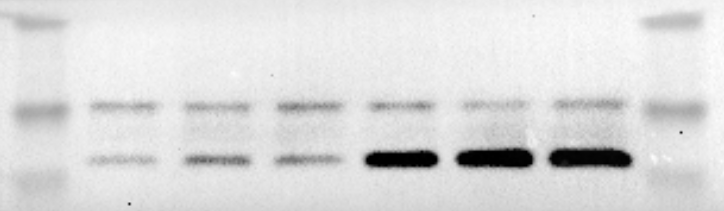

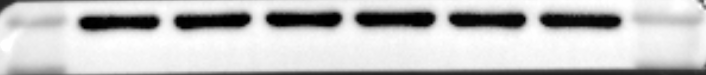

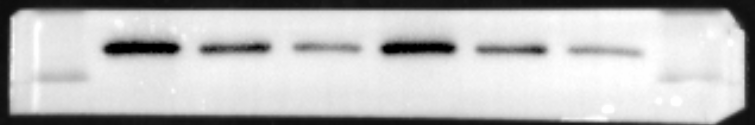


β-Actin

NNMT

-45kD

-29kD

P62

-62kD

LC3B I

LC3B II

-16kD

-14kD

**（4）Figure 3E**


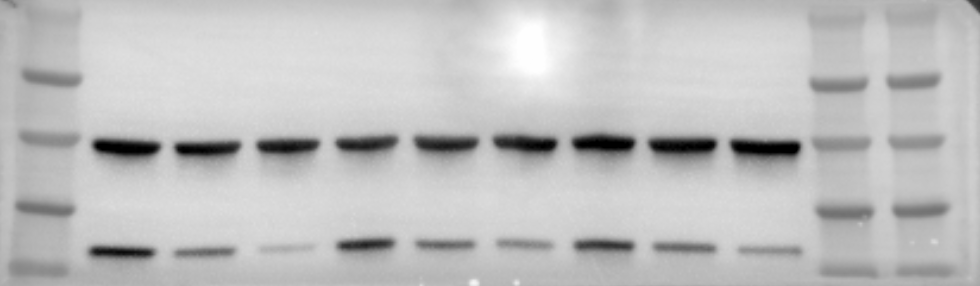

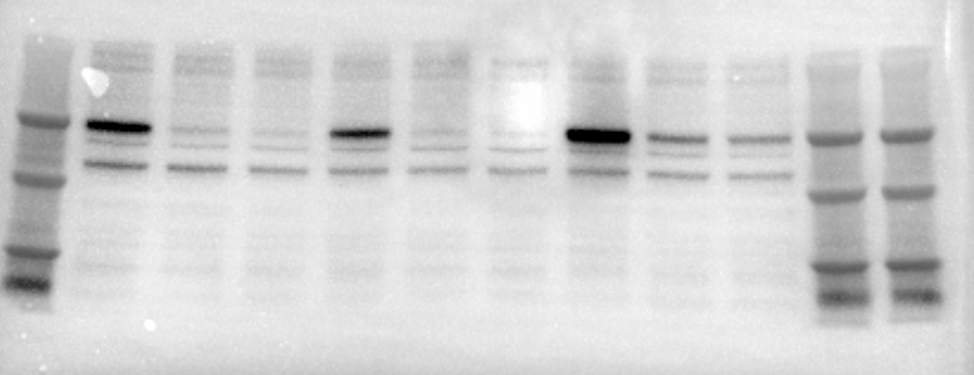


β-Actin

NNMT

-45kD

-29kD

-56kD

ALDH1A3

**（5）Figure 3F**

β-Actin

NNMT

-45kD

-29kD

-56kD

ALDH1A3


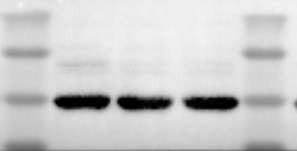

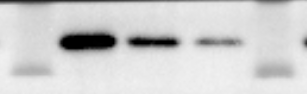


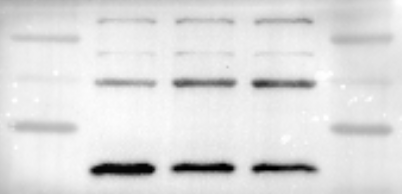

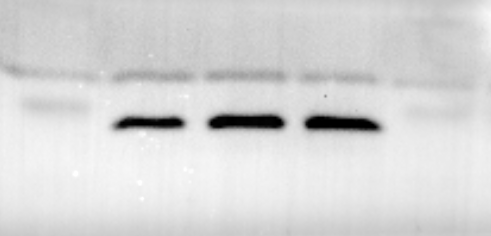

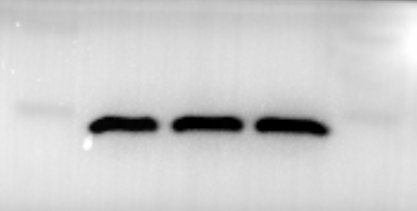

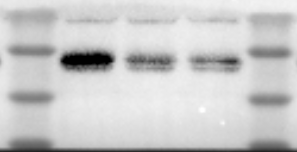
**（6）Figure 3G**

-15kD

NNMT

-29kD

H3K9me3

-15kD

H3

-45kD

**（7）Figure 3H**

-29kD

NNMT

H3K9me3

-15kD

-15kD

H3


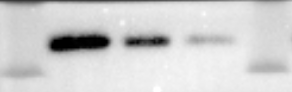


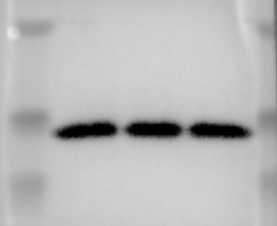

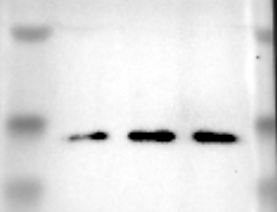


**（8）Figure 4C**


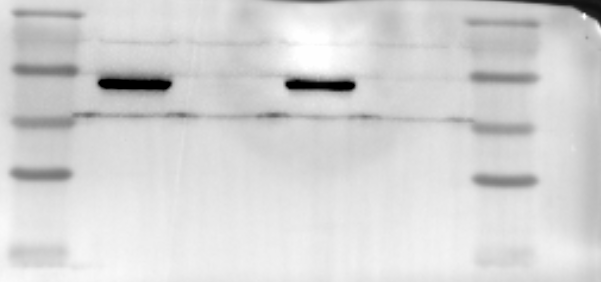

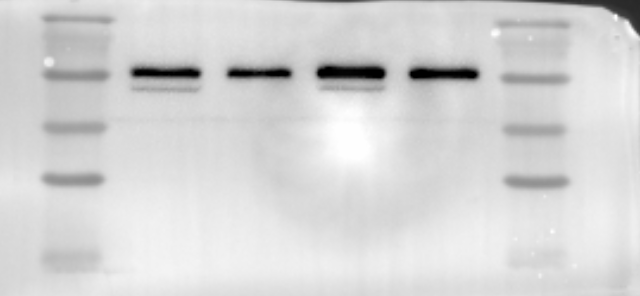

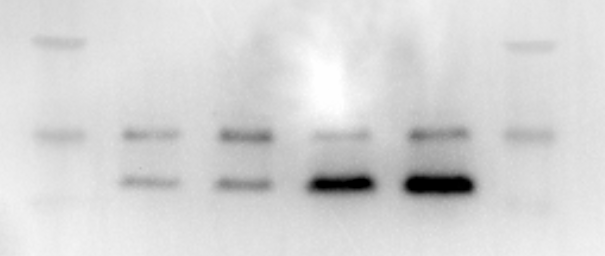


β-Actin

-45kD

-56kD

ALDH1A3


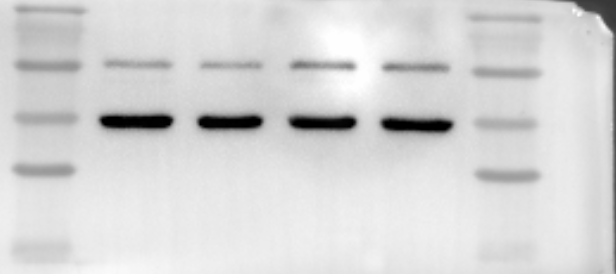


P62

-62kD

LC3B I

LC3B II

-16kD

-14kD


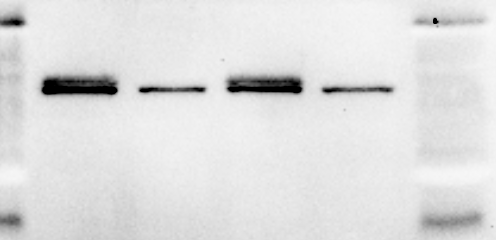
**（9）Figure 4D**

β-Actin

-45kD

-56kD

ALDH1A3

P62

-62kD

LC3B I

LC3B II

-16kD

-14kD


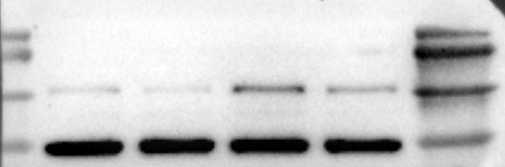

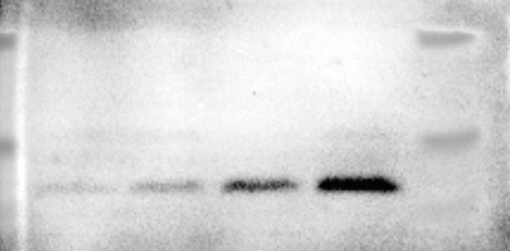

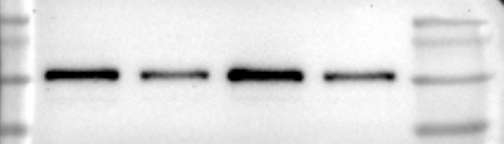


**（10）Figure 4I**


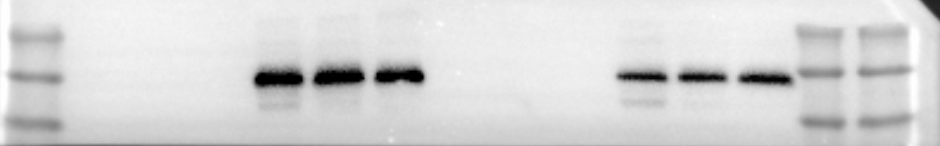

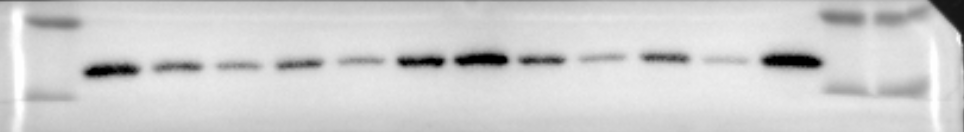

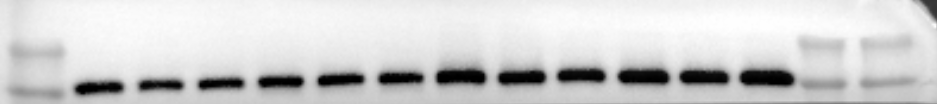

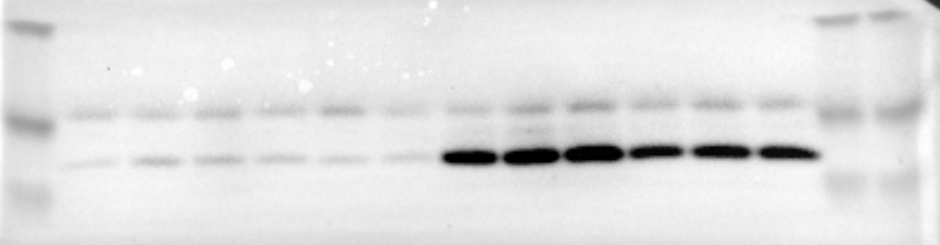

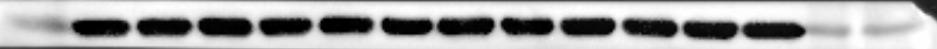


β-Actin

-45kD

-56kD

ALDH1A3

P62

-62kD

LC3B I

LC3B II

-16kD

-14kD

-29kD

NNMT

**（11）Figure 4J**


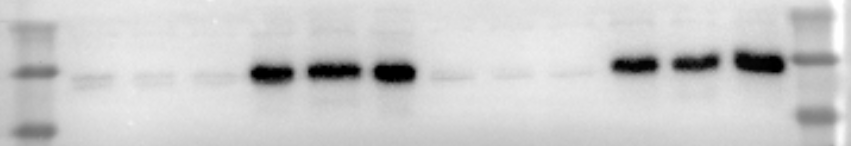

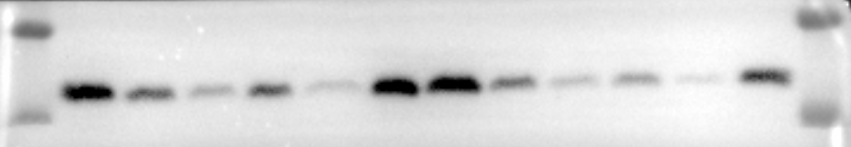


β-Actin

-45kD

-56kD

ALDH1A3

P62

-62kD

LC3B I

LC3B II

-16kD

-14kD

-29kD

NNMT


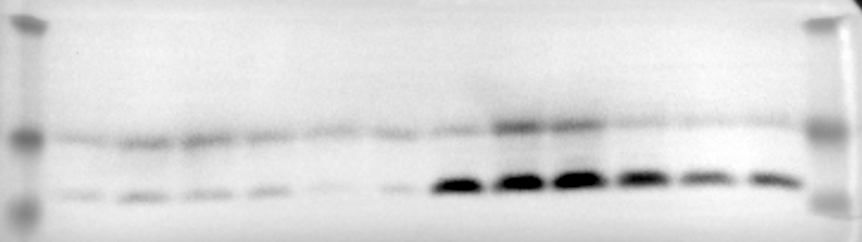

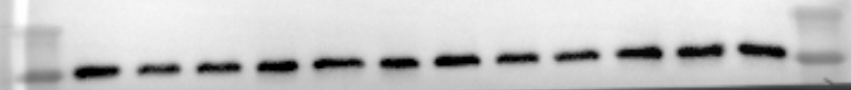


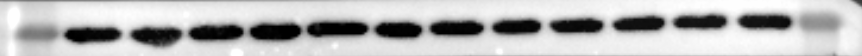


**(12) Figure 5D**


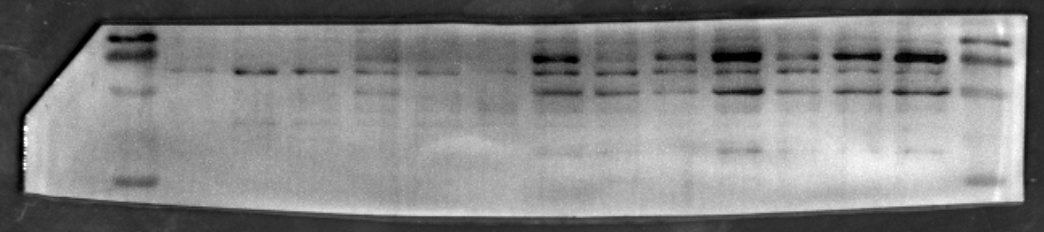

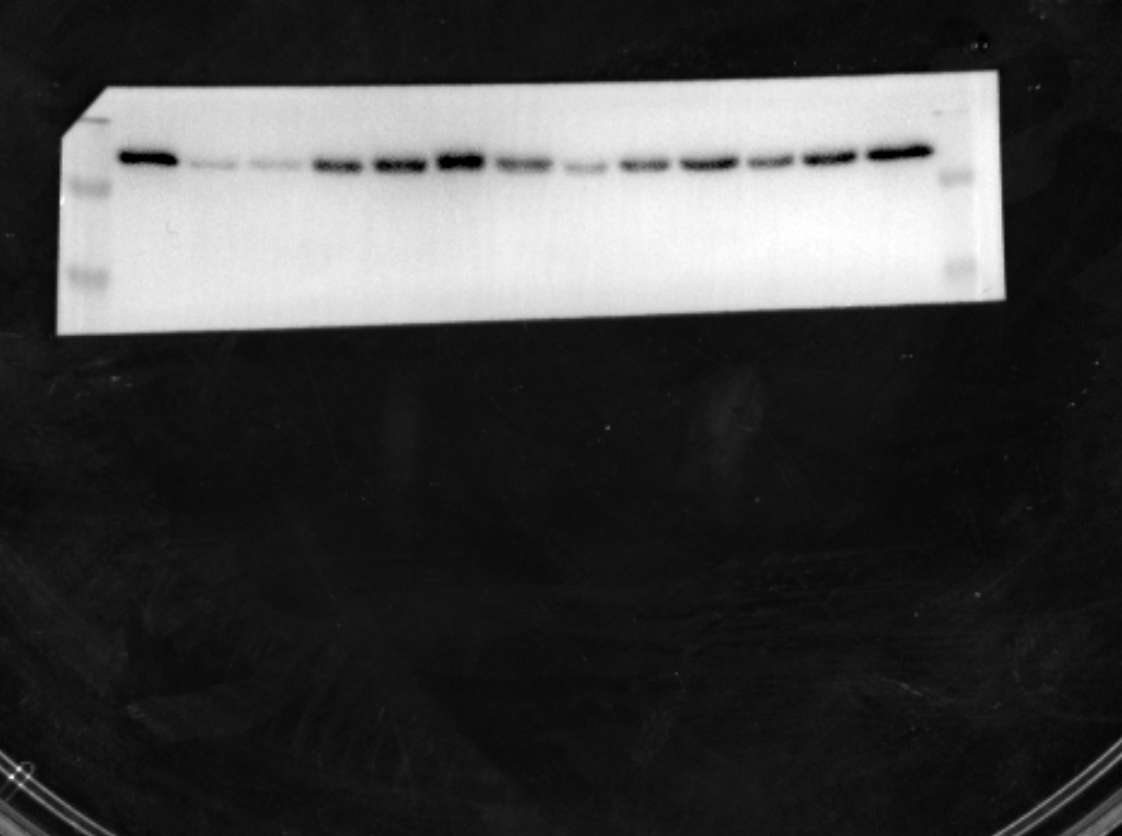

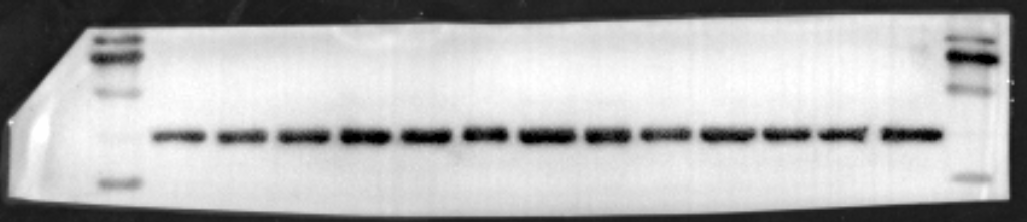


β-Actin

-45kD

-56kD

ALDH1A3

-29kD

NNMT

**
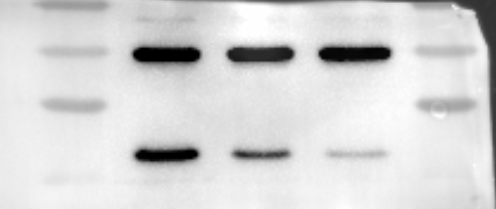
（13）Figure S1B**

-29kD

-45kD

NNMT

β-Actin


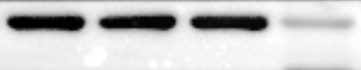

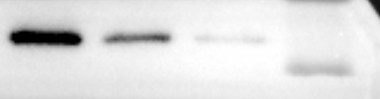
**（14）Figure S1D**

β-Actin

-45kD

-29kD

NNMT


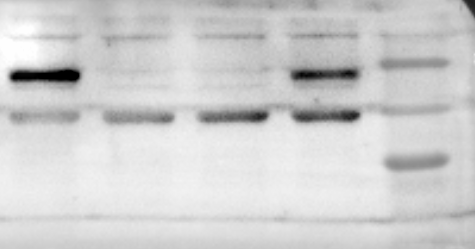
**（15）Figure S1H**

β-Actin

-45kD

-56kD

ALDH1A3

P. S. The four lanes correspond to NC (the first lane) and three siRNAs targeting ALDH1A3 (the subsequent three lanes). The first lane (the second lane from the left) was ultimately selected based on the phenotype.

**（16）Figure S1J**


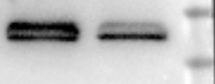


β-Actin

-45kD

-56kD

ALDH1A3


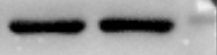

Supplement: Supplementary file 2 — Original images of western blot [file 41420_2025_2752_MOESM2_ESM.docx]
