# Supplementary figures and images for: Insufficient NNMT promotes autophagy and disrupts progesterone signaling in endometrial stromal cells in recurrent implantation failure by modulating the H3K9me3-ALDH1A3 pathway
Source: Cell Death Discov. 2025 Oct 7;11:450. doi: 10.1038/s41420-025-02752-x (PMC12504709; doi:10.1038/s41420-025-02752-x)

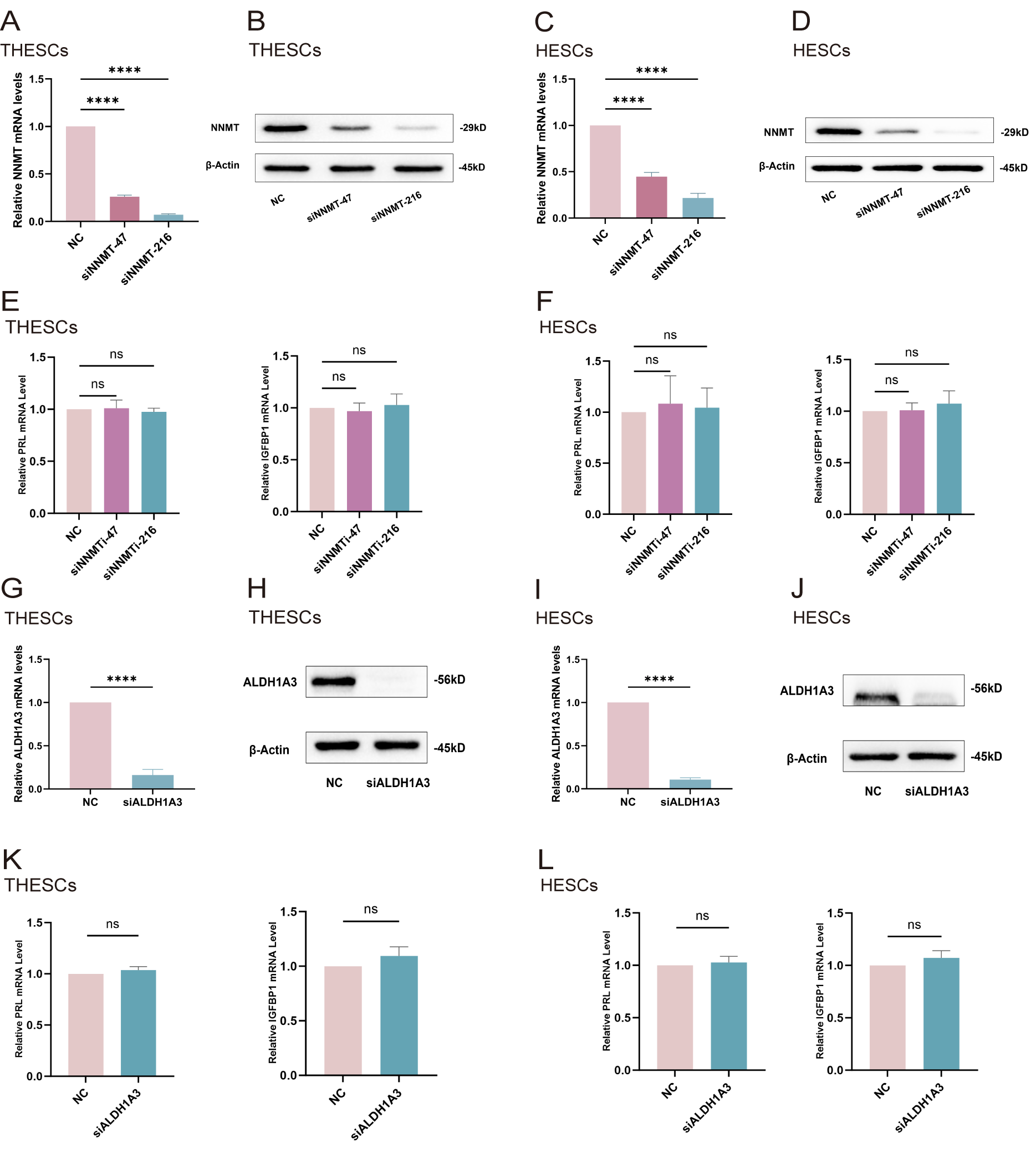

Supplement: Supplementary file 3 — Figure S1 [file 41420_2025_2752_MOESM3_ESM.tif]
